# Can the supplementary consumption of baobab (*Adansonia digitata *L*.*) fruit pulp improve the hemoglobin levels and iron status of schoolchildren in Kenya? Findings of a randomized controlled intervention trial

**DOI:** 10.1007/s00394-020-02447-2

**Published:** 2020-12-23

**Authors:** Esther Charlotte Evang, Tsige-Yohannes Habte, Willis Omondi Owino, Michael Bernhardt Krawinkel

**Affiliations:** 1 Institute of Nutritional Sciences, Justus Liebig University Giessen, Wilhelmstr. 20, 35392 Giessen, Germany; 2 School of Food and Nutritional Sciences, Jomo Kenyatta University of Agriculture and Technology, Juja, 62000-00200 Nairobi Kenya

**Keywords:** Baobab, Anemia, Bioavailability, Schoolchildren, Kenya

## Abstract

**Purpose:**

In the rural Kenyan diet, the bioavailability of iron is low and predisposes the population to iron deficiency. Fruit pulp of the indigenous baobab tree contains significant amounts of vitamin C, which enhances non-heme iron bioavailability. We studied the impact of baobab fruit pulp (BFP) consumption on the hemoglobin (Hb) and iron status of Kenyan schoolchildren.

**Methods:**

The single-blind randomized controlled intervention trial was implemented daily among apparently healthy schoolchildren aged 6–12 years with hemoglobin level < 12.2 g/dl. For 12 weeks, children in the intervention group (*n* = 29) received a drink with BFP, while the control group (*n* = 29) received an isoenergy drink without BFP. At baseline and endline, blood samples were taken.

**Results:**

The development of hemoglobin, ferritin (FER) and soluble transferrin receptor (sTfR) did not differ significantly between the intervention and control groups. However, in the intervention group, Hb levels improved slightly (2.2%), while they decreased slightly (1.2%) in the control group. Levels of geometric means of sTfR remained almost unchanged (0.7%) in the intervention group and slightly worsened (2.7%) in the control group. In both the groups, geometric mean of FER levels decreased, yet to a smaller extent in the intervention (17.3%) than in the control (26.0%) group.

**Conclusion:**

Even though no significant effects of BFP could be detected in this study, the identification of products such as BFP remains pertinent to help improve non-heme iron absorption in the most vulnerable populations.

## Introduction

In sub-Saharan Africa, anemia is widespread and associated with increased morbidity and mortality [1, 2], and impaired cognitive and behavioral development in children [3]. In Kenya, inadequate food intake is an important driver of anemia in schoolchildren [4], especially a combination of high intake of anti-nutrients [5] and low intake of heme iron [5, 6]. Other drivers of anemia are parasitic infections (*Plasmodium falciparum*, helminths and schistosomiasis) [4] and hemoglobin disorders [7, 8].

The latest representative data in Kenyan children aged 6 months to 12 years show an anemia prevalence of 25% [9], which is classified as moderate public health problem [10]. Nonetheless, a regional heterogeneity in the burden of anemia attributable to different etiological factors [4] has been confirmed by several studies. A study on schistosomiasis and soil-transmitted helminths in Kenyan schoolchildren reported 61% with anemia [11]. A recent study on the malaria risk among Kenyan children found 68.8% of the children studied to be anemic, with 23.6% affected by iron deficiency anemia [12]. Other studies representing regional differences reported prevalence of iron deficiency of 33%, 15% and 6.3% [5, 13, 14] and tissue iron deficiency from 20.3 to 70.4% [13].

In a rural population in Kenya, low iron bioavailability was found to be of greater concern than iron intake. Low intake of heme iron and high intake of phytate and polyphenols in the common diet [6] impair iron absorption. Non-heme iron by nature has a low bioavailability because it tends to crystalize in the small intestine; however, vitamin C increases its bioavailability [15]. Therefore, one approach in preventing anemia and iron deficiency is to improve the bioavailability of non-heme iron by increasing intake of vitamin C and other organic acids [16]. Vitamin C prevents the dose-dependent inhibitory effects of polyphenols and phytates on iron absorption [17] and further studies confirmed vitamin C to enhance non-heme iron bioavailability [18–20]. Positive associations between dietary vitamin C intake and hemoglobin (Hb) and ferritin (FER) levels have been found in Mexico, where a traditional beverage (*pulque*) containing 30 mg vitamin C is consumed with a diet based on cereals and beans [21].

The fresh fruit pulp of indigenous baobab trees *(Adansonia digitata *L*.)* contains a high amount (> 200 mg / 100 g) of vitamin C [22, 23], which is unparalleled compared to vegetables and other fruits [24]. In vitro studies on food-to-food fortification of cereal porridge with baobab fruit pulp (BPF) showed enhancement of iron bioaccessibility, probably because it is rich in both vitamin C and other organic acids such as citric acid [25–27]. Nnam et al. [28] studied the effect of vitamin C from BFP on hemoglobin levels and iron status of Nigerian schoolchildren over 3 months. They found a significant improvement in Hb and FER levels of schoolchildren that received a drink with BFP after a meal. Nnam et al. concluded that baobab pulp is a nutritious, natural and inexpensive source of vitamin C with positive implication on the iron status of Nigerian children.

Our *Baobab Nutrition Intervention Study* aimed to define the role of BFP in alleviating iron deficiency problems among schoolchildren in Kenya. The study was part of the BAOFOOD research project that studied the use, processing and market development of underutilized baobab for improved food and nutrition security and rural livelihoods in Kenya. The nutrient composition of BFP has been studied previously, unlike nutrition evaluation of BFP in terms of bioavailability [22]. BFP is locally available within the baobab belt in Kenya (one part in the inland from the Tanzanian border towards the north-east and a second one along the whole coastal region [29]). It is easily accessible to even the poorest communities, thereby offering a sustainable way to prevent micronutrient deficiencies [27]. The objective of the study was to determine the impact of BFP consumption on the Hb and iron status of Kenyan schoolchildren aged 6–12 years.

## Materials and methods

### Study design and sites

The study was performed as a single-blind placebo controlled, parallel group study [1:1]. Public primary schools were purposely selected according to the following criteria: (a) school meal program in place, (b) public day school, (c) at least 280 children aged 6–12 years, and (d) accessibility by car. The primary schools with comparatively large number of students in the study area where the authorities and the head teachers expressed support and were open for the intervention were approached.

Under these criteria, Kakumuti Pre- and Primary School was selected in rural Kitui-West (Sub-county), Kitui (County), Eastern Province of Kenya, approximately 165 km away from Nairobi. About 430 children attended the school, which had a self-governed school meal program. Kitui County is of marginal agricultural potential, prone to droughts [30] and the stunting prevalence in children under five is among the highest in the country [2]. Kitui is considered a low-risk area for malaria transmission [9].

Kitui belongs to the baobab belt [29], except of certain sub-counties such as Kitui-West where the few baobab trees do not produce fruits. Around the school, there were no baobab products identified in the markets, and baobab fruits used for the study were sourced from another area, namely Kyamatu location in Kitui-East Sub-county. The intervention started in May 2018, which is generally the end of the long rainy season. The average rainfall was above normal in 2018, and general food security improved during the intervention period, which fell in the postharvest season of staples and pulses.

### Sampling study participants

**Fig. 1:**
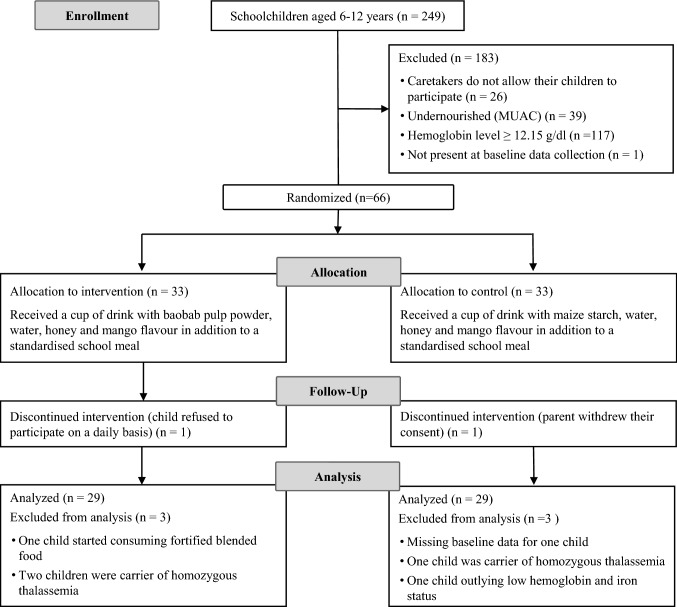
Consort flow diagram of the *Baobab Nutrition Intervention Study.* MUAC: mid-upper arm circumference

After obtaining official research permits and consent from the school administration, locally trained project assistants described the study in the local Kikamba language to caregivers of the children eligible for screening. Only children whose caregivers provided written informed consent (signature or fingerprint) were invited for the screening. The assistants orally informed these children about the study objective and procedure of the upcoming exercise, and children approved the reception of the information with their signature. Children’s oral consent and their signature were prerequisite for any further interview and examination. Registered nurses and laboratory technicians performed the clinical screening in a separate room and administered a dewormer (Albendazole USP 400) to all children. Thus, intestinal blood loss due to helminth infections was prevented. Eligible participants were apparently healthy children aged 6–12 years with lowest adjusted Hb level at screening. Exclusion criteria are shown in Fig. 1.

### Intervention

The intervention took place daily for a total of 83 days from May to July 2018. In addition to a standardized school meal, study children received either one cup of a drink with BFP or one cup of an isoenergy drink without BFP. The standardized portion of the school meal had an estimated iron content of 7.6 mg per portion, mainly from beans (NutriSurvey2007) (Table 1). BFP is rich in vitamin C [22, 31]; therefore, we expected an improvement in the bioavailability of iron from the school meal.

**Table 1:** Energy and nutrients of one portion of school meal (calculated in NutriSurvey^©^)

Variables	Portion = 456 g
Energy (kcal)	601
Vitamin C (mg)	1
Iron (mg)	7.6
Zinc (mg)	3.5
Calcium (mg)	105
Magnesium (mg)	201

The preparation of the school meal (mixed beans, maize, iodized salt, and vegetable oil) was standardized. Baobab fruits were delivered from Kyamatu and processed on a daily basis by trained local field assistants. They cleaned the fruits with a wire brush first and with a soft brush afterwards to remove the hair from the outer shell. The fruits were then cracked with a machete, and those with any spots (insects, mold, etc.) inside the fruit were discarded applying the two man rule. The pulp-seed mix was removed from the shell, ground in a mortar to separate pulp (which is in the form of a powder) from the seeds, and the powder sieved twice in succession.

About 20–30 min prior to distributing the drinks to the children, all ingredients for the intervention drink were blended. A weighted cup of intervention drink contained 20 g BFP, 5 g honey, 7 drops of Mango Liquid Flavour Drops (SygLabs, Germany), and 200 ml of bottled water. The isoenergy control drink consisted of 3 g commercially available corn starch, 10 g honey, 5 Mango Liquid Flavour Drops (SygLabs, Germany) and 220 ml of bottled water. The corn starch was boiled in 2 l of bottled water and mixed with the remaining ingredients after cooling. The field assistants weighed 220 g of either baobab drink or control drink in cups. The cups were coded with different colors to differentiate between intervention and control drink. Table 2 shows the nutrient composition of the intervention and control drink. The field assistants observed the children during the consumption to avoid any exchange of food and drinks and recorded the amount of food and drink consumed by each child.

**Table 2:** Energy and nutrition composition of 220 ml intervention and control drink (calculated in NutriSurvey^©^)

Variables	Intervention	Control
Energy (kcal)	40.2	41.2
Vitamin C (mg)	33.3	0
Iron (mg)	0.9	0.1
Zinc (mg)	0.6	0.1
Calcium (mg)	91.9	11.6
Magnesium (mg)	48.3	2.3

During the intervention, eight BFP samples were taken, stored in the fridge, and protected from light until a laboratory analysis was performed. The vitamin C was determined in triplicate using the method of Vikram et al. [32] with slight modifications. The samples was analyzed using a Shimadzu HPLC (20A Model, Tokyo, Japan), fitted with a ODS-C18 (250 cm × 4.6 mm × 5 µl) column, CTO-10AS VP oven, SPD-M20A diode array detector, DGU-20ASR prominence degassing unit, CBM-20A prominence communications bus module, SIL-20A HT prominence auto sampler and an LC-20AD prominence liquid chromatograph. The mobile phase contained 0.8% metaphosphoric acid at a flow rate of 0.8 ml/min. The injection volume used was 20 µl at a wavelength of 266 nm and oven temperatures of 30 °C. The retention time of pure ascorbic acid was used to identify ascorbic peaks in sample chromatographs. Iron, zinc, calcium, and magnesium were analyzed in duplicate with an inductive coupled plasma-optic emission spectrometer as described by Habte et al. [33]. Table 3 shows the BFP composition.

**Table 3:** Baobab fruit pulp composition

Variables (mg / 100 g)	Mean ± SD (*n *= 8)
Vitamin C	166 ± 71
Iron	4.1 ± 1.3
Zinc	2.7 ± 1.0
Calcium	408 ± 68
Magnesium	232 ± 77

### Allocation into the intervention and control groups

The allocation of participating children into either the intervention or control group was done using the stratified random sampling in SPSS. Participants were stratified according to sex (30 male and 36 female), Hb level above and below median for male (md = 11.9 g/dl) and female (md = 11.8 g/dl), respectively, resulting in four blocks. Among each block, a random allocation in intervention and control group was performed with the Mersenne Twister random number generator conducted in SPSS (V 24) according to age in years.

### Sample size

A total of 33 children were allocated into each group, with an assumed dropout of 10%, and a prevalence of homozygote and mixed forms of sickle cell disease and α-thalassemia of 6% (own data), and 76% of the children with Hb-levels > 11.5 g/dl [9]; we aimed to have data of 56 children be available at the endpoint. Given this sample size, we expected to detect medium to strong effects (Cohen’s d = 0.76) with alpha = 0.05 and power = 80, two-sided. The number of probands was expected to translate to 15% decrease in mean sTfR in the intervention group with an unchanged mean sTfR in the control group (mean baseline and control groups: 8.48; mean intervention group at endline: 7.208) with a standard deviation of 1.32 at both time points and a correlation of 0.25 between time points. These values were copied from Perignon et al. [34] as we did not have our own data when the study was planned; the assumed correlation of 0.25 is a conservative assumption.

We initially aimed to screen a total of 273 children but found 249 children aged 6–12 years only, of whom 223 children participated in the screening. When we selected the school, we were only given the numbers of children per class. Information on child age was provided only after the school had been selected.

### Blood sample collection and analysis

To minimize any discomfort, a local anesthetic ointment containing lidocaine and prilocaine (EMLA™, AstraZeneca, Cambridge, UK) was applied onto the area of skin to be numbed prior to pricking. During screening, capillary blood samples of children were taken for two subsequent Hb measurements using a HemoCue HB 301 photometer device (HemoCue AB, Ängelholm, Sweden). The maximum tolerated difference between the measurements was 0.5 g/dl. The mean value was used to determine individual Hb levels at screening.

At baseline, registered nurses took from each child a non-fasting venous blood sample, which was spun within 30 min to obtain 50–100 µl serum. The serum was pipetted into labeled 0.2 ml Multiply^®^ PCR tubes (Sarstedt Inc., US). In the field, samples were either stored at low temperature for a maximum of 7 days and then put into a freezer or stored in a freezer on the same day [35]. The samples were analyzed for serum ferritin (FER), soluble transferrin receptor (sTfR), acidic glycoprotein (AGP), and C-reactive protein (CRP) levels using a Sandwich ELISA at the VitMin Lab, Willstaett, Germany, [36]. Hb concentrations were measured immediately after phlebotomy using a HemoCue HB 301 photometer device (HemoCue AB, Ängelholm, Sweden).

Hb was adjusted for altitude and anemia, which is defined as adjusted Hb < 11.5 g/dl in children aged 7–11 years and < 12 g/dl in children aged 12 years [37]. Iron deficiency was defined by depleted iron stores (adjusted FER < 15 µg/L) [38] and tissue iron deficiency by high serum sTfR (> 8.3 mg/L) [36].

CRP and AGP were assessed for the identification and classification of inflammation: incubation (CRP levels > 5 mg/L and AGP levels ≤ 1 g/L), early convalescence (CRP levels > 5 mg/L and AGP levels > 1 g/L), and late convalescence (CRP levels ≤ 5 mg/L and AGP levels > 1 g/L). FER was adjusted for inflammation stage with correction factors for each inflammation stage [39].

Genotyping for sickle cell trait and the 3.7 kb α-globin deletion that most commonly causes α ^+^-thalassemia in African populations was conducted by PCR [40, 41] at the KEMRI-Wellcome Trust Research Laboratories in Kilifi, Kenya, as described in detail previously.

### Anthropometric measurements

At screening, nurses received an additional instruction on how to assess the mid-upper arm circumference (MUAC) with a measuring tape that allows for an assessment to the nearest 0.1 cm. Moderate undernutrition was defined at MUAC < 14.5 cm and < 18.5 cm for children aged 6–9 years and 10–12 years, respectively [42].

To control for a potential influence of anthropometric developments from baseline to endline, we assessed weight and height at baseline and endline. Children were checked for edema and weighed without shoes and in light clothing to the nearest 0.1 kg, using a Seca^®^ UNICEF scale (SECA 874, Hamburg, Germany). Body height was measured to the nearest 0.5 cm using a calibrated SECA^®^ height scale (SECA 213, Hamburg, Germany). Weight and height measurements were repeated twice with a maximum tolerable difference of 0.1 kg for weight and 0.5 cm for height.

The weight-for-age z-score (WAZ), body mass index-for-age z-score (BAZ), and height-for-age z-score (HAZ) were calculated using Anthro Plus, the anthropometric calculator module based on the 2007 WHO reference for children aged 5–19 years [43, 44]. Stunting, underweight, and thinness were defined by HAZ, WAZ, and BAZ below − 2 SD, respectively. The school provided data on the age of the children, which was crosschecked with primary caregivers. If the primary caregiver could not verify the date of birth, WAZ, BAZ, and HAZ were not calculated.

### Assessment of nutrient intake

To control dietary intake outside the study setting, we conducted 24 h recalls during the 1st (t1), 5th (t2) and 11th (t3) weeks. Interviewers with a formal qualification in nutrition or food science, as well as literate in English and the local language, were trained on applying standardized 24 h recalls with primary caregivers. The questionnaire and 24 h recalls were translated into the local Kikamba language and retranslated into English, reviewed during the 6-day interviewer training, pre-tested, and modified to ensure meaning equivalence of the questions. Pre-testing was carried out among households with children not involved in the study.

The interviews for the multiple pass 24 h recalls consisted of (a) listing all foods and drinks consumed the day before the interview, (b) gathering detailed information about each food or recipe for dishes, (c) estimated quantification of the amount of consumed food/drink and used ingredients for the recipes, and (d) reviewing the information with the respondent at the end of the recall. Specially designed photo books were developed to estimate the quantity of intake of food and drinks. The interviewer also used local measuring tools such as spoons and cups for quantifying portion sizes.

Table 4 shows the recommended dietary allowances for energy, vitamins, and trace elements for school-aged children. Individual energy adequacy ratios were calculated as total energy intake divided by sex, and age-specific energy requirements, based on the recommendations of the FAO/WHO/UNU expert committee on human energy requirements [45]. The nutrient adequacy ratio (NAR) was determined for vitamins C, iron, zinc, calcium, and magnesium. Individual NARs were calculated as a total intake of the nutrient divided by the recommended daily allowance (RDA) for that nutrient, based on intakes recommended by the Kenyan Ministry of Health [46]. Table 1 shows the energy and nutrients of one portion of school meal that was provided on a daily basis in addition to the drink.

**Table 4:** Recommended dietary allowances of energy, vitamin, minerals, and elements for school-aged children

Energy and nutrients^a^	6–8 years	9–12 years
Energy (kcal)	1694	1916
Vitamin C (mg)	25	45
Iron (mg)	10	8
Zinc (mg)	5	8
Calcium (mg)	800	1300
Magnesium (mg)	130	240

^a^Reference values of the Kenyan National Micronutrient Survey 2011 [2] except for energy (presented as means for boys/girls aged 6–8 years and 9–12 year, respectively) [30]

### Data management and statistical analysis

Data entry and validation via double entry was performed for anthropometry and Hb, as well as for the 24 h recalls. The country-specific food database for Kenya was loaded into the NutriSurvey nutrient database. Missing food items were supplemented from the Tanzania Food Composition Tables [47] and the Food Data Central of the United States Department of Agriculture [48].

Data management and statistical analysis were performed using SPSS software (Version 24, IBM Corp., Armonk, NY, USA).

The mean intake of energy and nutrients, determined through NutriSurvey, at time points t1, t2, and t3 was calculated for each child. Normality of distributions was evaluated using the Shapiro–Wilk test. As most continuous variables (micronutrient status and energy and nutrient intake) had heavily skewed distributions, descriptive statistics for continual variables are presented in the median and interquartile range (IQR). For this data, a non-parametric median test was applied for comparing data from intervention and control groups at baseline (blood parameters and anthropometric data) and at t1, t2, and t3 (mean energy and nutrient intake). The strength of association was calculated with Cramer’s V, which equals r. For approximately normally distributed data, means and standard deviations are presented, and the independent *t* test was applied.

Outliers in development (baseline to endline) of Hb, FER, and sTfR were identified as described by Tukey [49] and excluded from the analysis (outliers: n(Hb) = 0; n(FER-intervention) = 3, n(FER-control) = 1; n(sTfR-intervention) = 0, n(sTfR-control) = 1).

The baseline and endline data on FER and sTfR were log transformed and used to calculate the development between baseline and endline to apply the independent samples’ *t* test for differences between groups and the paired *t* test for development within the group. The effect size for the independent *t* test was not calculated (differences not significant) and paired *t* test was calculated using Cohen’s d.

Friedman’s ANOVA was conducted to test differences in dietary intake between t1, t2, and t3 (related samples and pairwise comparison). The general linear model was used to evaluate the effects of time (baseline/endline), group (intervention/control), age (in years at baseline), change in weight (endline—baseline), sex (male/female), and genotype (heterozygote carrier of α-thalassemia/non-carrier) on Hb, LN(FER), and LN(sTfR) and the interaction of time with each variable, respectively. For Hb, we also analyzed the interaction time*group*genotype. Variables were tested for associations with non-parametric Spearman’s correlation. A *p* value of < 0.05 was considered statistically significant.

### Ethical approval

The institutional review board of the Faculty of Medicine at Justus Liebig University Giessen, Germany (197/16) and the AMREF Ethics and Scientific Review Committee (AMREF- ESRC P313/2017) Kenya approved the *Baobab Nutrition Intervention Study* under the Kenyan National Commission for Science, Technology, and Innovation research permit (NACOSTI/P/18/60305/20841). The study was registered with the German Clinical Trials Registry (DRKS00011935). Official permission and approval from Kenya government authorities was obtained, and the municipal and governmental authorities in Kenya approved for the implementation of the study.

Written informed consent of primary caregivers and schoolchildren via signature or fingerprint was obtained prior to data collection. The ethics committees also approved the consent format prior to data collection. The management school board comprising the parent’s representative, representatives from the Kenya National Union of Teachers, church and local leaders were informed about the study and gave their verbal consent after participating in a stakeholder meeting to create awareness on the study.

## Results

Of the 249 eligible schoolchildren aged 6–12 years, a total of 223 were screened. After randomization, allocation, and follow-up, data of 58 children was available for the analyses. To include the required number of children, a cut-off for low Hb levels was set at 12.15 g/dl, i.e., 6% above the normal cut-off at 11.5 g/dl used in Kenyan schoolchildren otherwise. The intervention lasted for 83 days, and the median days of participation was 82 in both groups (IQR intervention: 78–82.5 and IQR control: 79–83).

In both groups, 55.2% of participants were girls, and 37.9–41.1% of participants were heterozygous carriers of α-thalassemia in the intervention and control group, respectively. None of the participants were carriers of sickle cell trait.

### Baseline characteristics

At baseline, median test did not show significant differences between intervention and control group in terms of median Hb (non-adjusted and adjusted), FER (non-adjusted and adjusted), sTfR, CRP and AGP. Elevated inflammation markers were only present in the control group, with prevalence of 1, 1, and 3 children in incubation, early convalescence, and late convalescence, respectively. Prevalence of low Hb-, FER-, and sTfR levels are presented in Table 5.

**Table 5:** Hemoglobin level and iron status at baseline and endline

	Intervention		Control	
	Baseline	Endline	Baseline	Endline
Hemoglobin (g/dl)	*n* = 29		*n* = 29	
Hb, mean ± SD	12.6 ± 0.72	12.9 ± 0.87	13.0 ± 0.69	12.9 ± 0.10
Hb, adj^a^ mean ± SD	12.4 ± 0.72	12.7 ± 0.87	12.8 ± 0.69	12.7 ± 0.10
Hb, adj^a^ < 11.5, * n* (%)	2 (6.9)	2 (6.9)	1 (3.4)	2 (6.9)
FER (µg/L)	*n* = 26		*n* = 28	
FER, geometric mean ± SD	36.7 ± 1.89	30.7 ± 1.96	42.7 ± 1.96	30.2 ± 1.78
FER adj^b^, geometric mean ± SD	36.7 ± 1.89	30.4 ± 1.96	40.1 ± 1.90	29.7 ± 1.78
FER adj^b^, < 15 µg/L, *n* (%)	4 (15.4)	3 (10.3)	4 (14.3)	3 (10.7)
sTfR (mg/L)	*n* = 29		*n* = 28	
sTfR geometric mean ± SD	6.27 ± 1.21	6.31 ± 1.19	6.16 ± 1.21	6.33 ± 1.21
sTfR > 8.3, *n*(%)	2 (6.9)	3 (10.3)	2 (7.1)	3 (10.7)

*Hb* hemoglobin, *FER* Ferritin, *sTfR* soluble transferrin receptor

^a^Hemoglobin adjusted for altitude

^b^Ferritin adjusted for inflammation stage

Table 6 shows the baseline characteristic of the anthropometric measurements. In the intervention group, the prevalence of underweight, stunting, and thinness was 21.7%, 23.1%, and 3.8% and in the control group the prevalence was 15.0%, 13.4%, and 3.4%, respectively.

**Table 6:** Mean (SD) of anthropometric characteristics for intervention and control group at baseline

	*n*	Intervention	*n*	Control	*p*
		Mean ± SD		Mean ± SD	(*t* test)
Age					
Age in years	29	8.2 ± 1.8	29	8.7 ± 1.9	0.259
Nutritional status					
Weight (kg)	29	22.8 ± 5.5	29	24.8 ± 5.7	0.177
Height (cm)	29	123.0 ± 10.0	29	126.8 ± 11.1	0.174
WAZ	26	− 1.4 ± 0.9	29	− 1.3 ± 0.8	0.611
HAZ	26	− 1.2 ± 1.0	29	− 1.1 ± 0.8	0.739
BAZ	26	− 0.9 ± 0.7	29	− 0.7 ± 0.8	0.549

Date of birth was not confirmed for three rural children and nutritional status was not calculated

*WAZ* weight-for-age z-score, *HAZ*: height-for-age z-score, *BAZ* body mass index-for-age z-score

### Dietary intake of meals in and outside the school

Median energy and nutrient intake (calculated from mean individual intakes at t1, t2, and t3 for each child) are presented in Table 7, as well as median adequacy ratio. Median vitamin C and calcium intake was significantly higher in the intervention group with a medium effect size (vitamin C: *χ*^2^(1) = 11.655, *p* = 0.001, *r* = 0.448; calcium: *χ*^2^(1) = 8.345, *p* = 0.004, *r* = 0.379). A critical nutrient was calcium because the actual intake was far below recommendations, with a median adequacy ratio of 39% (IQR = 32–52%) in the intervention and 30% (IQR = 25–35%) in the control group.

**Table 7:** Median (IQR) daily intake and median adequacy ratio for intervention and control group

	Median (IQR) daily intake		*p* (median test)	Median adequacy ratio	
	Intervention (*n* = 29)	Control (*n* = 29)		Intervention (*n* = 29)	Control (*n* = 29)
Energy (kcal)	1633 (1403–1829)	1524 (1359–1721)	0.189	92%	83%
Vitamin C (mg)	73 (57–96)	40 (27–63)	0.001	211%	122%
Iron (mg)	15 (13–17)	14 (13–15)	0.066	165%	157%
Zinc (mg)	10 (8–11)	8 (8–9)	0.066	156%	128%
Calcium (mg)	396 (344–477)	318 (276–364)	0.004	39%	30%
Magnesium (mg)	513 (435–581)	437 (402–492)	0.066	310%	242%

**Fig. 2:**
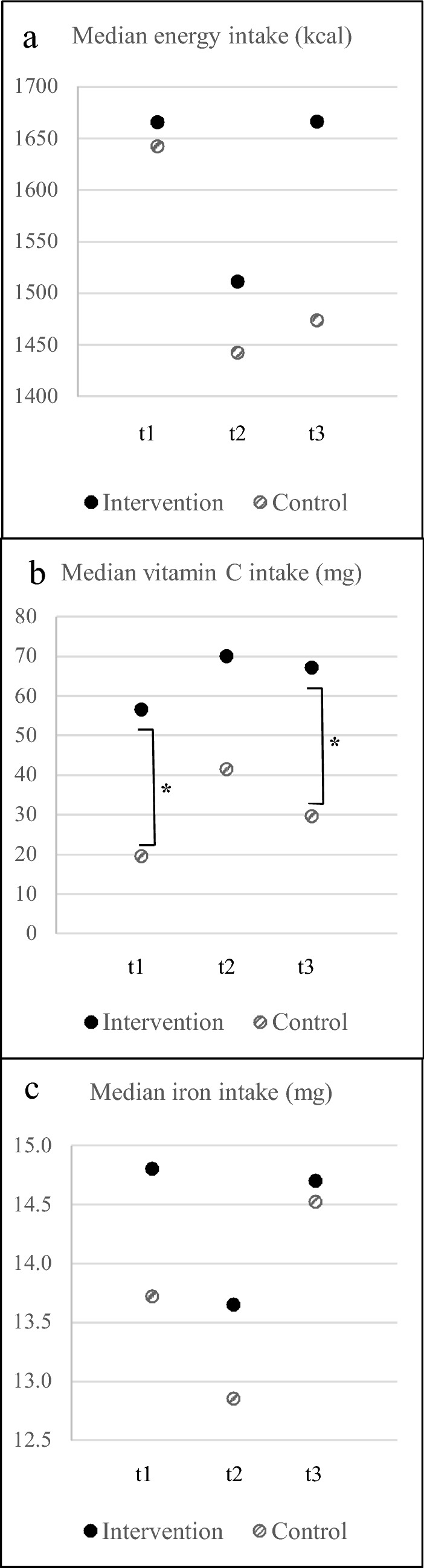
Median intake of energy, vitamin C and iron at t1, t2 and t3 of the intervention (*n* = 29) and control (*n* = 29) group. *Significant differences between intervention and control group (*p* < 0.05)

During the intervention, the dietary intake changed (Fig. 2). At t2, vitamin C intake increased, while energy and iron intake decreased compared to t1. At all times, median adequacy ratio for iron was met in both groups (≥ 140%) and the intake of vitamin C and iron at t1, t2, t3 did not differ significantly in the intervention and control group, respectively. Only the energy intake in the control group differed significantly between t1 and t2 with a small effect size (*p* = 0.026, *r* = 0.128). Noteworthy, the energy intake was always higher in the intervention than in the control group.

### Impact of baobab intake on hemoglobin and iron status

**Fig. 3:**
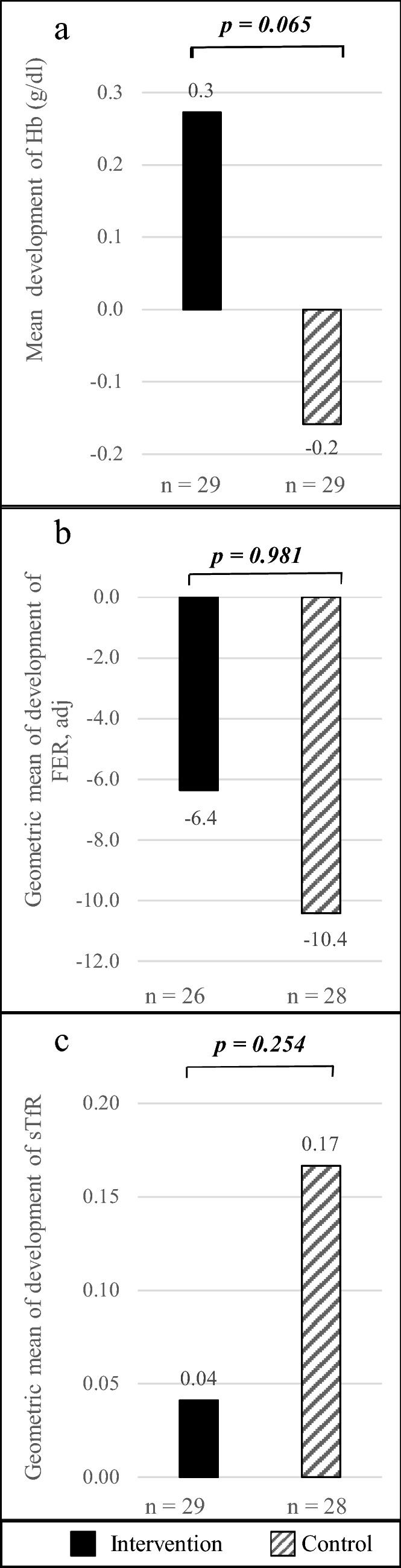
Changes in hemoglobin and iron status from baseline to endline; independent *t* test was applied for difference in development between intervention and control group. **a** Mean development of Hb with non-significant differences between intervention and control group. **b** Development of geometric mean of FER and with non-significant difference of LN(FER) development between intervention and control group. **c** Development of geometric mean of sTfR with non-significant difference between development of LN(sTfR) in the intervention and control group. Hb: hemoglobin, FER: ferritin adjusted, sTfR: soluble transferrin receptor

Table 5 shows the baseline and endline data of the intervention and control group without outliers in development. Figure 3 presents the changes in Hb, FER, and sTfR in both groups. Between baseline and endline, developments showed a better tendency of Hb (mean), FER (geometric mean), and sTfR (geometric mean) in the intervention than in the control group. Hb and sTfR within each group did not significantly change between baseline and endline. Mean Hb levels improved by 2.2% in the intervention and worsened by 1.2% in the control group. Geometric mean of sTfR level in the intervention group levels remained almost unchanged (0.7%) while it worsened by 2.7% in the control group. The number of children with tissue iron deficiency increased by one in each group between baseline and endline.

Geometric mean of FER levels worsened by 17.3% in the intervention and 26.0% in the control group. Mean LN(FER) values within the intervention *t*(27) = 3.820, *p* = 0.001, *r* = 0.675) and control (*t*(25) = 3.444, *p* = 0.002, *r* = 0.722 group significantly worsened. Even though the mean FER level significantly decreased in both groups, the number of children with iron deficiency decreased by one child in each group.

The general linear model including age, change of weight, group, gender, carrier of heterozygous α-thalassemia and did not show a significant influence on changes in Hb, LN(FER), or LN(sTfR). Nonetheless, we found small evidence of an effect on the group on Hb (part. Eta^2^ = 0.067; *p* = 0.062).

## Discussion

Positive associations between dietary vitamin C intake and Hb levels, as well as iron status, have been found in Mexico, where a traditional beverage (*pulque)* containing 30 mg vitamin C is consumed with a diet based on cereals and beans. Although the diet was high in phytate and phenolic compounds, similar to the diet in the *Baobab Nutrition Intervention Study*, a higher vitamin C intakes predicted a lower risk of anemia [21]. A study on the impact of BFP intake of Nigerian schoolchildren found that a BFP-drink can significantly improve Hb-levels and iron stores. The amount of vitamin C from the BFP in the intervention drink was twice as high as in the *Baobab Nutrition Intervention Study* (60 mg vs. 33 mg vitamin C), the control group did not receive any placebo and the prevalence of anemia and iron deficiency was much higher in the Nigerian study [28]. The intervention drink was consumed before the meal with similar ingredients (cereal/legume/vegetable-based meal) as in the *Baobab Nutrition Intervention Study*, but detailed information on food are not provided. Furthermore, information on randomization, blinding, food composition or dietary intake of participants are missing. Though, in vitro studies with BFP are in line with the Nigerian study as they found significant improvements iron bioaccessibility, probably due to the rich vitamin C content and other organic acids such as citric acid [25–27].

The observations from this study can be summarized as follows: mean Hb levels slightly increased in intervention group and slightly went down in controls, but both changes were not statistically significant. The geometric mean of FER levels went significantly down in both groups, but to a lower extent in the intervention than in the control group. Geometric mean of sTfR levels increased in the intervention group and more markedly increased in controls. Overall, the whole study population experienced a general tendency towards worsening iron stores. The tendencies of changes are concordant and point towards a beneficial effect of BFP on iron absorption as summarized in Table 8. Since the study population had an unexpectedly low prevalence of anemia and iron depletion, significant effects could not be demonstrated within the chosen study design. Although the schoolchildren were selected for low to low normal Hb levels, the observed change—brought about the consumption of BFP as a supplement to the school meal—were not as high as expected.

**Table 8:** Tendencies of the findings in both groups

	Intervention	Control
Hb	↑	↓
FER	↓	↓↓
sTfR	↑	↑↑

To control for the intervention effect, we selected a low-risk area for malaria transmission, we provided albendazole to children at screening, and determined the two most common Hb-disorders, sickle cell and α-thalassemia trait. Therefore, it is unlikely that worsening of FER levels can be attributed to helminth infections. Moreover, α-thalassemia trait was neither associated with baseline Hb, FER, and sTfR levels nor with their development, while sickle cell trait was not present in the studied population.

The expected intervention effect of vitamin C on improved iron bioavailability might have been mitigated by inhibitory compounds of the school meal and the BFP itself. To allow for a significant enhancing effect of iron absorption, Teucher et al. [15] suggest a molar ratio of 2:1 and of 4:1 of vitamin C to iron for meals with low medium and high levels of inhibitors, respectively. In the *Baobab Nutrition Intervention Study* the calculated molar ratio for the vitamin C rich BFP and iron of the phytate rich school meal was lower, namely 1.3:1. Besides compounds in BFP that promote the iron bioavailability, BFP also contains phenolic compounds that are generally known to inhibit iron absorption. However, of the total phenolics, 21.5% were identified as catechin [26], a strong promoter of iron bioavailability [50]. But, other phenolics found in BFP are iron-chelating compounds, in particular tannins [25, 26, 31]. The tannin content may be caused by contamination of the fruit pulp with seed fragments, which themselves are high in tannins [51]. The BFP in our study was processed by mechanical separation of the pulp from the seeds using a mortar. Even though we sieved the BFP two times, we cannot exclude contamination with seed fragments.

During the intervention, the experienced food security improved (data not presented here) as the intervention started at the end of the rainy season. This was reflected in a change of dietary intake, towards a higher intake of vitamin C and lower energy and iron intake towards the middle of the intervention (t2) (Fig. 2). Due to the positive dose-depended relationship of vitamin C intake and iron bioavailability [52], the lower iron intake could have been compensated by higher iron bioavailability. However, FER levels decreased in both groups during the intervention even though the iron intakes above the recommended intake. Notably, presented changes of vitamin C intake were attributed to dietary patterns at household level, because the composition of the intervention drink and school meal remained unchanged throughout the intervention. However, the impact of BPF on improved bioavailability of iron might have varied widely, according to the natural variations of the vitamin C content of the BFP ranging from 80 to 266 mg/100 g. In conclusion, the low iron bioavailability may have been the limiting factor for iron utilization in this study.

Apart from the tested intervention effect, we found significantly higher vitamin C and calcium intake in the intervention than in the control group. In BFP, both nutrients are particularly high (mean vitamin C and calcium intake through BFP: 33 mg and 81 mg, respectively). An inhibitory effect of calcium on iron absorption has been discussed in several studies; yet, a review on long-term calcium supplementation concluded that there is no adverse effect on iron status [53]. Moreover, a 1-month calcium supplementation did not result in a reduction of iron bioavailability [54].

BPF contains iron, zinc, magnesium, and phosphorous; however, the higher intake of these nutrients in the intervention than in the control group was not at a significant scale (*p* = *0.066*, respectively). However, the energy intake in the intervention groups was higher, yet not significantly, scale, which may also partly explain the higher intake of nutrients. Nevertheless, the energy intake from intervention and control drink was equivalent (Table 2).

### Limitations

The prevalence of anemia and iron deficiency in this study population of Kenyan schoolchildren was much lower than expected; therefore, the intervention effect was also lower. The study was conducted in a non-malaria-endemic zone, which might partly explain the lower anemia prevalence compared to other studies [5, 11, 12, 14]. As the sample size was calculated on the assumption of a higher prevalence of iron deficiency, the actual sample size was too small to show significant effects on anemia and iron status.

The measured vitamin C content of the raw BPF for the intervention drink varied widely (Table 3). Therefore, the impact of BPF on the bioavailability of iron might have varied from day to day.

## Conclusion

In vitro studies showed an increased bioaccessibility of iron from cereals in the presence of the comparable amounts of BFP that were used in our study. However, in vivo, we detected a BFP-driven tendency towards better iron uptake from plant foods, but a significantly improved iron status brought about by supplementation with BFP could not be detected. We conclude that the promoting effect on iron bioavailability from BFP might not have overcome the inhibitory effect of phytate and polyphenols from the school meal. Adverse effects of BFP consumption have not been observed. The identification of products such as BFP remains pertinent to help improve non-heme iron absorption in the populations most vulnerable for iron deficiency. This is particularly relevant for food insecure areas where baobab is native, available, and affordable. Thus, school meal programs that include iron-rich foods as well as components promoting iron uptake are a reasonable approach to prevent childhood anemia.

We suggest to conduct a similar study in a setting with higher prevalence of anemia and to provide a fermented iron-rich cereal porridge (sorghum, etc.) mixed with BFP as the present study did not exclude the expected benefits.
